# Retraction: Seeding the future of nanomaterials: a comprehensive review of nanosheet-mediated growth for energy harvesting, energy conversion, and photodetection applications

**DOI:** 10.1039/d6ra90107g

**Published:** 2026-08-18

**Authors:** Attia Shaheen, Nadeem Raza, Irfan Ijaz, Aysha Bukhari, Mavra Farrukh, Mostafa E. Salem

**Affiliations:** a Henan Key Laboratory of High-Temperature Functional Materials, School of Materials Science and Engineering, Zhengzhou University Zhengzhou 450001 China; b Department of Chemistry, College of Science, Imam Mohammad Ibn Saud Islamic University (IMSIU) Riyadh Kingdom of Saudi Arabia; c School of Chemistry, Faculty of Basic Sciences and Mathematics, Minhaj University, Lahore Lahore 54700 Pakistan iffichemixt266@gmail.com; d Institute of Micro-nanoscale Optoelectronics, Shenzhen University Shenzhen 518060 China

## Abstract

Retraction of ‘Seeding the future of nanomaterials: a comprehensive review of nanosheet-mediated growth for energy harvesting, energy conversion, and photodetection applications’ by Attia Shaheen *et al.*, *RSC Adv.*, 2025, **15**, 20469–20494, https://doi.org/10.1039/D5RA01655J.

The Royal Society of Chemistry hereby wholly retracts this *RSC Advances* article as an institutional investigation by Zhengzhou University confirmed that the authors did not have permission to publish this article and the listed authorship is incorrect.

Signed: Laura Fisher, Executive Editor, *RSC Advances*

Date: 11th August 2026

